# Immune-Related LncRNAs to Construct a Prognosis Risk-Assessment Model for Gastric Cancer

**DOI:** 10.3390/curroncol29070391

**Published:** 2022-07-12

**Authors:** Shilin Zhi, Bin Yang, Shengning Zhou, Jianan Tan, Guangyu Zhong, Fanghai Han

**Affiliations:** Department of Gastrointestinal Surgery, Sun Yat-sen Memorial Hospital, Sun Yat-sen University, Guangzhou 510120, China; zhishl@mail2.sysu.edu.cn (S.Z.); yangb23@mail.sysu.edu.cn (B.Y.); zhoushn3@mail.sysu.edu.cn (S.Z.); tanjn3@mail.sysu.edu.cn (J.T.); zhonggy6@mail.sysu.edu.cn (G.Z.)

**Keywords:** gastric cancer, immune infiltration, lncRNA

## Abstract

Background: Gastric cancer is a prevalent cause of tumor death. Tumor immunotherapy aims to reshape the specific immunity to tumors in order to kill the tumor. LncRNAs play a pivotal role in regulating the tumor immune microenvironment. Herein, immune-related lncRNAs were used to establish a prognosis risk-assessment model for gastric cancer and provide personalized predictions while providing insights and targets for gastric cancer treatment to enhance patient prognosis. Methods: Gastric adenocarcinoma transcriptome and clinical data were acquired from the The Cancer Genome Atlas (TCGA) database to screen the immune-related lncRNAs. Then, LASSO COX regression was utilized to construct the prognosis risk-assessment model. Afterward, the reliability of the model was evaluated the relationship between immune infiltration, clinical characteristics, and the model was analyzed. Results: We identified 13 lncRNAs and constructed the prognosis assessment model. According to the median risk score of the training set, the patients were assigned to different risk groups. Overall survival time was shorter in the high-risk group. In the high-risk group, higher infiltration of mono-macrophages, dendritic cells, CD4+ T cells, and CD8+ T cells was observed. Moreover, the model was positively related to tumor metastasis. Conclusion: The prognosis risk-assessment model developed in this research can effectively predict the prognosis of gastric cancer patients. This tool is expected to be further applied to clinics in the future, thus providing a novel target for immunotherapy in gastric cancer patients.

## 1. Introduction

According to GLOBOCAN 2020, gastric cancer ranked fifth in terms of incidence and fourth in mortality among all cancers worldwide. The incidence of gastric cancer varies significantly by gender and region. The incidence in men is twice that in women. East Asia and East Europe have the highest incidence, especially in Mongolia, Japan, South Korea, and China, with an age-standardized rate (ASR) higher than 20 new cases per 100,000 [1]. Although the incidence and mortality of gastric cancer have considerably declined in the past few decades, its overall survival rate remains low. The 5-year age-standard survival rate of gastric cancer patients in the United States is 42.9% [2], while that of China is merely 27.4% [3]. Surgery is currently the gold standard for gastric cancer treatment, supplemented by neoadjuvant chemoradiotherapy or adjuvant chemoradiotherapy. They have improved the prognosis, but the outcomes are still not satisfactory. With the gradual application of tumor immunotherapy in clinical practice, researchers are actively exploring tumor immunotherapy in gastric cancer, which is anticipated to bring hope to gastric cancer patients. Tumor immunotherapy modifies the body’s specific immunity to tumors to eradicate them. Moreover, earlier studies have reported that immune-checkpoint blockade (ICB) therapy such as PD-1/PD-L1 improves the overall prognosis of gastric cancer patients [4,5].

Long noncoding RNAs (lncRNAs) are a type of RNA with a length of more than 200 nucleotides and do not encode functional proteins. Multiple studies have established that lncRNAs play an instrumental role in tumors. It has been reported that lncRNA is related to the loss of regulation of gastric cancer. Yang et al. corroborated that the lncRNA H19 was significantly upregulated in gastric cancer tissues and could partially inactivate P53 [6]. Sun et al. determined that HOXA11-AS promoted the invasion of gastric cancer cells and reduced the expression of KLF2 and PRSS8 by binding to RNA-binding proteins [7]. Meanwhile, lncRNAs have also been found to play a various of roles in the tumor immune microenvironment and can promote tumor immune escape by enhancing the sensitivity of T cells to activation-induced cell death [8]. They could influence the expression of downstream oncogenes and the recruitment of macrophages to promote tumor metastasis [9]. LncRNA-MM2P regulates tumor M2 macrophage polarization [10]. Owing to their rich biological functions, lncRNAs are considered novel biomarkers for gastric cancer. Moreover, lncRNAs have been used to construct reliable prognostic prediction models of tumor immune infiltration [11,12].

Gastric adenocarcinoma accounts for more than 90% of gastric cancers [13]. In the present study, transcriptome and clinical data of the TCGA project were used to construct a prognosis risk-assessment model for gastric adenocarcinoma based on immune-related lncRNAs, and to assess the novel immune-related lncRNA signature, including its predictive performance and the association with tumor immune infiltration and immune checkpoint genes. We aimed to provide personalized predictions while exploring the immune landscape of the model and providing ideas for gastric cancer treatment. 

## 2. Materials and Methods

### 2.1. Data Acquisition and Processing

The RNA-sequence (Fragments Per Kilobase per Million mapped reads, FPKM) and the clinical information of gastric cancer patients were collected from the TCGA-STAD project (https://portal.gdc.cancer.gov, accessed on 28 December 2020). In total, 343 gastric adenocarcinoma (GC) and 30 adjacent healthy tissues were included. Following this, the RNA-sequence was annotated with the GTF file acquired from the Ensembl project (http://asia.ensembl.org/index.html, accessed on 3 Febuary 2021) to identify the lncRNAs and mRNAs.

### 2.2. Immune-Related lncRNA Acquisition

The immune-related mRNAs were identified by combining the immune-related gene lists from ImmPort (https://www.immport.org/home, accessed on 24 January 2021) and our mRNA matrix. Then immune-related lncRNAs were screened by a coexpression strategy, which means a correlation analysis between immune-related mRNAs and all lncRNAs. LncRNAs with an absolute value of correlation coefficients higher than 0.4 and *p* < 0.001 were considered immune-related lncRNAs. Afterward, the R limma package was used for differential analysis of the immune-related lncRNAs between the GC and healthy group. LncRNAs with |logFC| > 1 and FDR (False Discovery Rate) < 0.05 were identified as differentially expressed immune-related lncRNAs (DEirlncRNAs).

### 2.3. Prognosis Risk-Assessment Model Construction and Validation

To improve the robustness of the model, the whole samples were randomly divided into a training set and a validation set (7:3). In the training set, univariate analysis was performed with *p* < 0.05 as the threshold to screen the prognosis immune-related lncRNAs. Secondly, Cox proportional hazard regression analysis following LASSO regression with 10-fold cross-validation was performed to construct the risk-assessment model. The risk score based on the model was calculated by the following formula: $Risksore=\sum Coefficient_{i}\;*\;Expression_{i}$. The area under curve (AUC) of time-dependent receiver operating characteristic curves (ROC) at 1, 3, and 5 years were plotted. The median risk score of the training set was used as the cutoff point to divide the GC patients into high-risk and low-risk groups. Kaplan–Meier analysis was subsequently used to analyze the difference in overall survival (OS) between the two groups, and verified in validation set and overall set using the R packages pheatmap and survivalROC.

### 2.4. The Associations between the Model and Immunity

The online database TIMER (http://timer.cistrome.org/, accessed on 24 January 2021) contains immune infiltration abundances of all TCGA samples estimated by six immune deconvolution methods, including TIMER [14], CIBERSORT [15], QUANTISEQ [16], XCELL [16], MCPcounter [17], and EPIC [18] algorithms. We extracted immune information of the patients in our study from the database; Spearman correlation analysis was performed to explore the association between the risk score and infiltration, with *p* < 0.05 considered statistically significant. Meanwhile, the abundance of immune-cell infiltration was compared between the two risk groups using Wilcoxon signed-rank test (*p* < 0.05). Lastly, the gene expression of immune checkpoints was compared in different risk groups using the R package ggpubr. 

### 2.5. Function-Enrichment Analysis of the Model

Kyoto Encyclopedia of Genes and Genomes (KEGG) pathway analysis with the computational method gene-set-enrichment analysis (GSEA) [19] was conducted in the whole set using the R packages clusterProfiler and enrichplot to compare the difference of pathways in the two risk groups.

### 2.6. Analysis of the Associations between the Model and Clinical Characteristics 

The multiple-factor ROC containing the model and TNM stage, tumor stage, grade, age, and gender was plotted to appraise the performance of the model. The Chi-square test was used to explore the association between the model and clinical characteristics (*p* < 0.05), and the results were illustrated on a band diagram. Wilcoxon signed-rank test was performed to analyze the difference in risk score in the clinical characteristics subgroups (*p* < 0.05), and the results were depicted as box drawings. To verify whether the model could be an independent prognostic predictor, both univariate and multivariate Cox regression analyses were performed (*p* < 0.05). The process was performed using R package ggplot2, pheatmap, and survivalROC.

**Figure 1:**
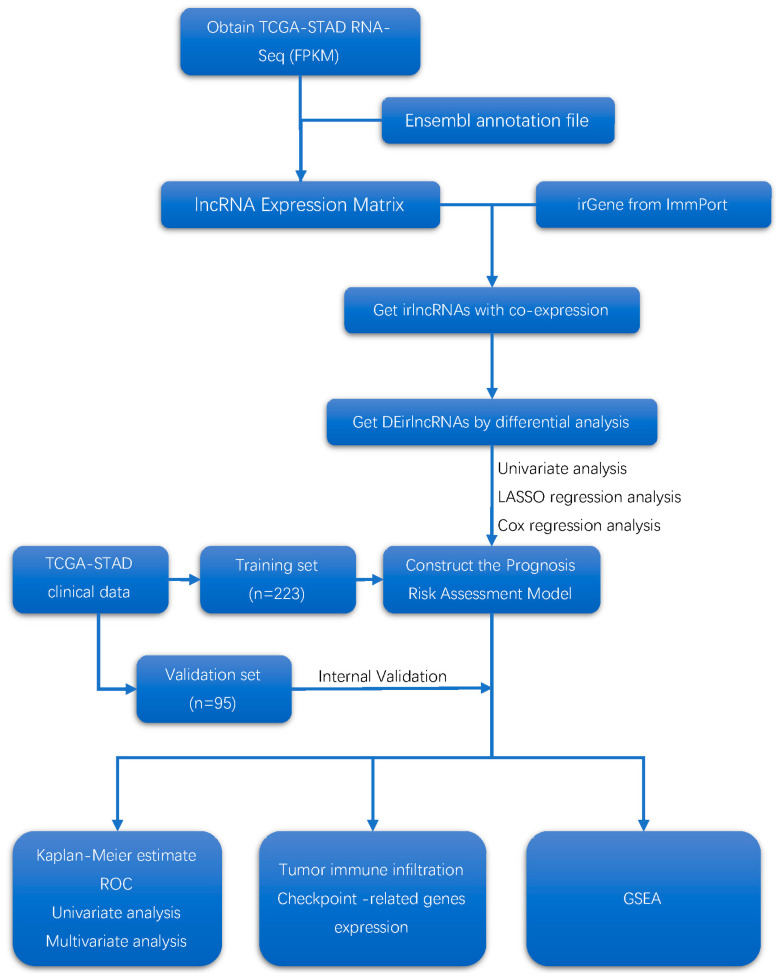
Flow diagram of the study.

The analytical procedure is shown in Figure 1.

## 3. Results

### 3.1. Screening Differentially Expressed Immune-Related lncRNAs

1082 immune-related lncRNAs were out screened by the coexpression strategy. And 414 differentially expressed immune-related lncRNAs were screened using differential analysis.

### 3.2. Construct and Validation the Prognostic Risk-Assessment Model

Patients lacking survival time were excluded, and clinical information of 318 patients was retained. The enrolled patients were randomly divided into a training set (*n* = 223) and a validation set (*n* = 95).

**Figure 2:**
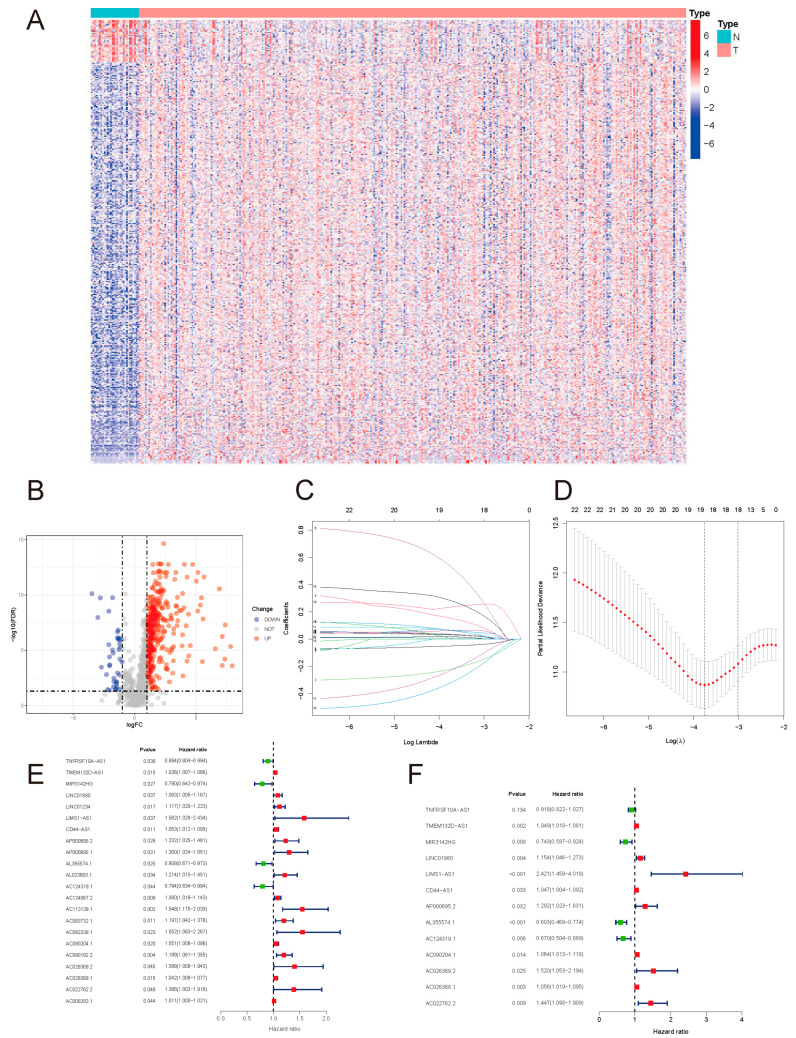
(**A**) DEirlncRNA heatmap. (**B**) DEirlncRNA volcano map. A total of 414 DEirlncRNAs were identified, including 375 upregulated and 39 downregulated ones. (**C**) Coefficients of the LASSO regression. (**D**) Partial-likelihood deviances of the LASSO model. (**E**) Prognostic DEirlncRNA forest map. (**F**) Forest map of lncRNAs included in the model.

Univariate analysis was performed for all DEirlncrnas in the training set and 22 prognostic lncRNAs were screened: TNFRSF10A-AS1, TMEM132D-AS1, MIR3142HG, LINC01980, LINC01234, LIMS1-AS1, CD44-AS1, AP000695.2, AP000695.1, AL355574.1, AL023803.1, AC124319.1, AC124067.2, AC113139.1, AC093732.1, AC092338.1, AC090204.1, AC090192.2, AC026369.2, AC026368.1, AC022762.2, and AC009283.1. Among them, 18 lncRNAs with a hazard ratio > 1 were risk factors promoting gastric cancer death. The remaining ones with a hazard ratio < 1, namely TNFRSF10A-AS1, MIR3142HG, AL355574.1 and AC124319.1, were considered protective factors for the prognosis of gastric cancer patients Figure 2E.

Based on these 22 lncRNAs, LASSO COX regression analysis was performed, and 13 lncRNAs were further identified: TNFRSF10A-AS1, TMEM132D-AS1, MIR3142HG, LINC01980, LIMS1-AS1, CD44-AS1, AP000695.2, AL355574.1, AC124319.1, AC090204.1, AC026369.2, AC026368.1, and AC022762.2, and the prognostic risk-assessment model was then constructed for gastric cancer Figure 2F. The lncRNAs used to construct the model are presented in Table 1.

The risk score was calculated as follows:

$$Risksore=\sum Coefficient_{i}\;*\;Expression_{i}$$

**Figure 3:**
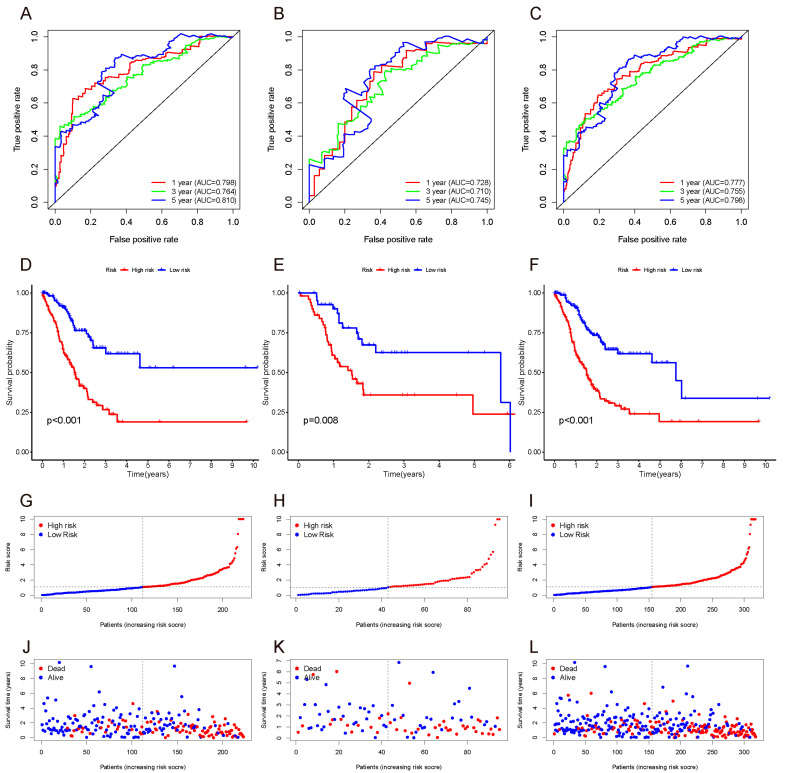
ROC (**A**–**C**), survival curve (**D**–**F**), risk score (**G**–**I**), and survival outcome (**J**–**L**) of the training set, validation set, and whole set.

The 1-, 3-, and 5-year ROC of the training set, validation set, and whole set were then plotted. The AUC values were all over 0.7, indicating a moderate potential for predicting prognosis (Figure 3A–C). In all three sets, the AUC at 5 years was the highest, indicating that the predictive performance at 5 years was superior to that at 1 year and 3 years.

We divided the GC patients into high-risk and low-risk groups based on the median risk score of the training set and performed Kaplan–Meier analysis to analyze the differences between the two groups. The results indicated that patients in the high-risk group had a worse prognosis than those in the low-risk group. The results were subsequently verified in the validation set and whole set (Figure 3D–F).

### 3.3. The Correlation between Risk Score and Immune-Cell Infiltration

**Figure 4:**
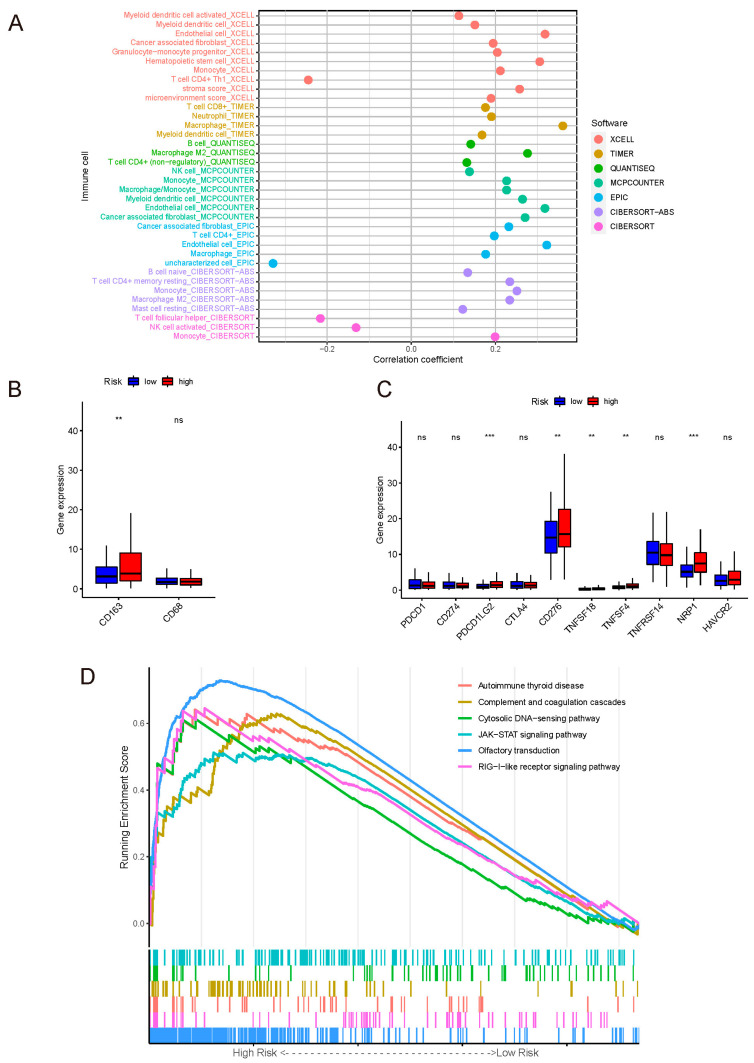
(**A**) Correlation coefficient dot plot of immune-cell infiltration abundance between the model and algorithms. Mono-macrophage system was positively correlated with the risk score in various algorithms. (**B**) Gene-expression levels of macrophages’ markers. Label: *p* < 0.01 = **, ns = not significant. (**C**) Gene-expression levels of immune check points. Label: *p* < 0.001 = ***, *p* < 0.01 = **, ns = not significant. (**D**) GSEA of the whole set.

Spearman correlation analysis was performed between the risk score of the whole set and the abundance of immune-cell infiltration. As displayed in Figure 4A and Table S1, the results suggested that the risk score was positively correlated with a variety of immune-infiltrating cells (*p* < 0.05), among which the mono-macrophages were the most significant. At the same time, the abundance of various types of immune-cell infiltration was compared between the risk groups (Table S2), again validating that mono-macrophages exhibited higher abundance in the high-risk group, followed by dendritic cells, CD4+ T cells, and CD8+ T cells. The results of comparison with *p* < 0.05 are illustrated in Figure S1; *p* values are shown on the line between the two groups. To further demonstrate the distribution of M2 macrophage phenotype, the gene-expression level of the M2-specific biomarker CD163 and pan-macrophage marker CD68 were compared between the two risk groups, and the result revealed that the M2 phenotype had a higher abundance in the high-risk group (Figure 4B).

The immune checkpoint is the core of immune therapy for malignant tumors. On one hand, our model was positively related to PD-L2, CD276, TNFSF18, TNFSF4, and NRP1. On the other hand, there was no correlation between our model and the common checkpoint biomarkers PD-1, PD-L1, and CTLA-4 (Figure 4C).

The result of GSEA for KEGG was shown in Figure 4D.

### 3.4. The Associations between Risk Score and Clinical Characteristics

**Figure 5:**
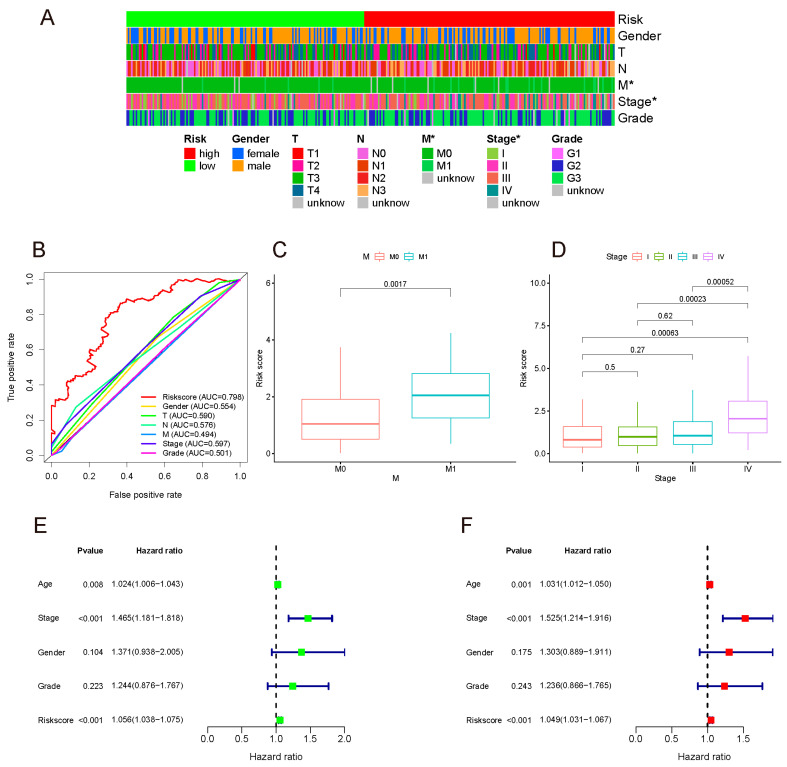
(**A**) Band diagram outlining the distribution of clinicopathological characteristics in the whole sample sorted by a risk score ranging from low to high. Label: *p* < 0.05 = *. (**B**) 5-year ROC comparing the performance of different signatures. Our model outperformed the others. (**C**,**D**) M stage and tumor stage were positively related to the risk score. Univariate (**E**) and multivariate (**F**) analyses confirmed that the model could be used to independently predict indicator.

The Chi-square test was performed on clinical data and risk groups (Table S3), and their relationship was analyzed and presented in the form of a band diagram (Figure 5A). The risk was related to the M stage and tumor stage. Thereafter, Wilcoxon signed-rank test results demonstrated that the M1 group had a higher risk score than the M0 group. The risk score of stage IV gastric cancer was also higher than that of other stages, and there was no statistical difference in pairwise comparison of STAGE I, II, and III, implying that the model may be more closely related to the metastasis of advanced tumors, as outlined in Figure 5C,D.

In the above studies, the AUC of our model at 5 years was highest, which was 0.798 for the whole set. The 5-year ROC of the whole set showed favorable prognostic prediction performance among the TNM stage, tumor stage, pathological grade, age, and gender (Figure 5B).

In order to identify whether the model was an independent prognostic indicator, univariate and multivariate COX analyses were performed. Due to the correlation between the TNM and tumor stage, only the latter was included in the analyses. Both univariate and multivariate analyses authenticated that this model could be used as an independent prediction indicator (Figure 5E,F).

## 4. Discussion

In recent years, the incidence of gastric cancer has significantly declined globally, which can be attributed to examination methods, prompt treatment, and early intervention of precancerous lesions. However, due to its insidious onset, most patients are diagnosed with moderate advanced gastric cancer, which has a poor prognosis. Therefore, there is an urgent need to identify a more personalized predictive signature.

In this study, immune-related lncRNAs were identified through correlation analysis from the TCGA-STAD database. To reduce the variability of single indicator, constructing a signature with appropriate indicators would be more reliable [20]. LASSO Cox regression analysis was conducted to construct a multifactor model with a reduced set of variables. Various methods were applied to evaluate the model, including ROC and K-M survival analysis, which determined that the model displayed satisfactory performance in distinguishing between different risk groups of patients to effectively predict the prognosis of gastric cancer patients. Indeed, our model could more accurately predict the outcome compared to other prevalent clinicopathological markers. Univariate and multivariate Cox analysis support the model as an independent signature for prognosis prediction. The above-mentioned tests reveal that our model we constructed is reliable and accurate.

The appraisal of associations between the risk score and immune infiltration suggested that the mono-macrophages, dendritic cells, CD4+ T cells, and CD8+ T cells had a higher abundance of infiltrations in the high-risk group, especially mono-macrophages.

The mono-macrophage infiltration significantly increased as the risk score increased. Macrophages have a tremendous influence on tumor growth [21]. Studies have revealed that macrophages exhibit an M1 phenotype in the early stage of tumor development, releasing inflammatory mediators and acting as antitumor agents [22,23]. As the tumor progresses, under hypoxic conditions in the tumor microenvironment, macrophages gradually shift to an M2 phenotype, exerting anti-inflammatory effects and promoting T-cell apoptosis [24,25]. Most of the algorithms we used in our studies seldom analyzed the phenotype of cells. We postulated the higher infiltration of M2 macrophages in the high-risk group, which plays a role in promoting tumor growth. Furthermore, the higher gene expression of the M2-specific biomarker CD163 in the high-risk group confirmed our assumption.

Contrary to the studies that high infiltration of CD8+ T cells brought a better tumor outcome [26], previous studies have reported that high infiltration of CD8+ T cells leads to a poor prognosis in GC patients [27], which is consistent with our study. The prognosis value of CD8+ T cells is worth further studies.

There were no significant differences in the expression of common checkpoint genes such as PD-1, PD-L1, and CTLA-4 between the two risk groups, which signified that our model could not predict the therapeutic effect of existing PD-1/PD-L1 or even CTLA-4 immune therapy. Actually, only about 15% PD-L1 positive patients responded to the PD-1/PD-L1 therapy [28], which limited the appliance of the therapy. However, the expressions of PD-L2 and some novel checkpoint genes were upregulated in the high-risk group, and studies have uncovered that PD-L2 leads to immune suppression and a poor prognosis in gastric cancer patients [29,30,31]; this may be the underlying immunotherapeutic target of our model.

According to the GSEA results, these pathways were upregulated in the high-risk group: olfactory transduction, RIG-I-like receptor signaling pathway, autoimmune thyroid disease, complement and coagulation cascades, cytosolic DNA-sensing pathway, JAK-STAT signaling pathway, neuroactive ligand–receptor interaction, and cytokine–cytokine receptor interaction. Complement and coagulation cascades and cytosolic DNA-sensing pathways participated in the mediation of the innate immune system [32,33]. We speculate our model is related to immune injury. The cytosolic DNA-sensing pathway can induce apoptosis through cGAMP binding to STING, commonly considered important to immune surveillance [34]. However, the cytosolic DNA-sensing pathway is also a double-edged sword and controversial. On one hand, the majority of researchers consider that tumor DNA activates the pathway to induce tumor-antigen presentation and activate the antitumor effector [35,36,37]; on the other hand, it releases vast inflammatory mediators to recruit M2 macrophage polarization, which we assume is what happened in our study [34,38].

Some lncRNAs in the signature are implicated in tumor development. It was previously reported that LINC01980 is overexpressed in esophageal squamous-cell carcinoma and acts as an endogenous competing RNA to regulate the downstream pathway and promote the development of esophageal cancer [39,40]. Chateauvieux et al. found that overexpression of MIR3142HG in myeloid leukemia prolongates patient survival, possibly by interrupting NF-κB signaling [41]. In our model, MIR3142HG was also found to be a prognostic protective factor for gastric cancer patients. WEI et al. identified TNFRSF10A-AS1 as a prognostic protective factor of colorectal cancer [42]. Studies on the majority of lncRNAs in our model are limited, and their role in gastric cancer warrants further investigations.

A major limitation of our study is that it was a single-center retrospective study, and external data were not validated; prospective studies are necessary to obtain a higher level of evidence. Another shortcoming of this study is the lack of in vivo and in vitro experiments to verify the robustness of the model. Therefore, clinical samples should be collected for further experimental studies in the future.

## 5. Conclusions

The immune-related lncRNA prognostic risk-assessment model for gastric cancer can effectively predict the prognosis of gastric adenocarcinoma patients. This model is expected to be applied in clinical practice to improve patients’ outcomes.

## Figures and Tables

**Table 1:** LncRNAs of the model.

lncRNA	Coefficient	HR	95% CI	*p* Value
TNFRSF10A-AS1	−0.085	0.918	0.822–1.027	0.134
TMEM132D-AS1	0.048	1.049	1.018–1.081	0.002
MIR3142HG	−0.298	0.743	0.597–0.924	0.008
LINC01980	0.143	1.154	1.046–1.273	0.004
LIMS1-AS1	0.884	2.421	1.459–4.018	0.001
CD44-AS1	0.046	1.047	1.004–1.092	0.033
AP000695.2	0.256	1.292	1.023–1.631	0.032
AL355574.1	−0.506	0.603	0.469–0.774	<0.001
AC124319.1	−0.401	0.670	0.504–0.889	0.006
AC090204.1	0.062	1.064	1.013–1.118	0.014
AC026369.2	0.419	1.520	1.053–2.194	0.025
AC026368.1	0.054	1.056	1.019–1.095	0.003
AC022762.2	0.370	1.447	1.098–1.909	0.009

## Data Availability

The data presented in this study can be found in the TCGA database (https://portal.gdc.cancer.gov). The GTF file can be acquired from the Ensembl project (http://asia.ensembl.org/index.html). The immune-related gene lists can be found in ImmPort (https://www.immport.org/home). And the immune infiltration information can be found in TIMER (http://timer.cistrome.org).

## Supplementary Materials

The following supporting information can be downloaded at: https://www.mdpi.com/article/10.3390/curroncol29070391/s1, Table S1. Correlation between risk score and abundance of immune cells. Table S2. P value of comparison of immune cells in the two risk groups. Table S3. Relationship between the risk groups and clinical characteristics. Figure S1. The result of the comparison of immune-infiltrating cells in the two risk group.
