# Supplementary material for: Immune-Related LncRNAs to Construct a Prognosis Risk-Assessment Model for Gastric Cancer
Source: Curr Oncol. 2022 Jul 12;29(7):4923–35. doi: 10.3390/curroncol29070391 (PMC9318354; doi:10.3390/curroncol29070391)
Supplement: Supplementary file 1 [file curroncol-29-00391-s001.zip › curroncol-1742171-supplementary.pdf]

Supplementary Materials

Immune-related LncRNAs to Construct a Prognosis Risk-Assessment Model for Gastric Cancer

Shilin Zhi , Bin Yang, Shengning Zhou, Jianan Tan, Guangyu Zhong and Fanghai Han

Table S1. Correlation between risk score and abundance of immune cells.

Immune Cells and Algorithms	The Correlation Coefficient	*p* Value
T cell CD8+_TIMER	0.176	0.002
Neutrophil_TIMER	0.191	<0.001
Macrophage_TIMER	0.361	<0.001
Myeloid dendritic cell_TIMER	0.168	0.003
T cell follicular helper_CIBERSORT	−0.216	<0.001
NK cell activated_CIBERSORT	−0.131	0.019
Monocyte_CIBERSORT	0.199	<0.001
B cell naive_CIBERSORT-ABS	0.134	0.017
T cell CD4+ memory resting_CIBERSORT-ABS	0.234	<0.001
Monocyte_CIBERSORT-ABS	0.251	<0.001
Macrophage M2_CIBERSORT-ABS	0.234	<0.001
Mast cell resting_CIBERSORT-ABS	0.122	0.029
B cell_QUANTISEQ	0.141	0.012
Macrophage M2_QUANTISEQ	0.277	<0.001
T cell CD4+ (non-regulatory)_QUANTISEQ	0.132	0.019
NK cell_MCPCOUNTER	0.139	0.014
Monocyte_MCPCOUNTER	0.227	<0.001
Macrophage/Monocyte_MCPCOUNTER	0.227	<0.001
Myeloid dendritic cell_MCPCOUNTER	0.265	<0.001
Endothelial cell_MCPCOUNTER	0.318	<0.001
Cancer associated fibroblast_MCPCOUNTER	0.271	<0.001
Myeloid dendritic cell activated_XCELL	0.113	0.044
Myeloid dendritic cell_XCELL	0.151	0.007
Endothelial cell_XCELL	0.318	<0.001
Cancer associated fibroblast_XCELL	0.195	<0.001
Granulocyte-monocyte progenitor_XCELL	0.205	<0.001
Hematopoietic stem cell_XCELL	0.306	<0.001
Monocyte_XCELL	0.212	<0.001
T cell CD4+ Th1_XCELL	−0.245	<0.001
stroma score_XCELL	0.258	<0.001
microenvironment score_XCELL	0.190	<0.001
Cancer associated fibroblast_EPIC	0.232	<0.001
T cell CD4+_EPIC	0.198	<0.001
Endothelial cell_EPIC	0.322	<0.001
Macrophage_EPIC	0.177	0.002
uncharacterized cell_EPIC	−0.329	<0.001

Table S2. P value of comparison of immune cells in the two risk groups.

Immune cell	*p* Value	Immune Cell	*p* value
B cell_TIMER	0.016	T cell CD4+ memory activated_CIBERSORT-ABS	0.854
T cell CD4+_TIMER	0.004	T cell follicular helper_CIBERSORT-ABS	0.67
T cell CD8+_TIMER	<0.001	T cell regulatory (Tregs)_CIBERSORT-ABS	0.239
Neutrophil_TIMER	0.002	T cell gamma delta_CIBERSORT-ABS	0.421
Macrophage_TIMER	<0.001	NK cell resting_CIBERSORT-ABS	0.856
Myeloid dendritic cell_TIMER	<0.001	NK cell activated_CIBERSORT-ABS	0.716
B cell naive_CIBERSORT	0.416	Monocyte_CIBERSORT-ABS	<0.001
B cell memory_CIBERSORT	0.284	Macrophage M0_CIBERSORT-ABS	0.946
B cell plasma_CIBERSORT	0.628	Macrophage M1_CIBERSORT-ABS	0.02
T cell CD8+_CIBERSORT	0.749	Macrophage M2_CIBERSORT-ABS	<0.001
T cell CD4+ naive_CIBERSORT	0.331	Myeloid dendritic cell resting_CIBERSORT-ABS	0.527
T cell CD4+ memory resting_CIBERSORT	0.276	Myeloid dendritic cell activated_CIBERSORT-ABS	0.129
T cell CD4+ memory activated_CIBERSORT	0.927	Mast cell activated_CIBERSORT-ABS	0.009
T cell follicular helper_CIBERSORT	0.011	Mast cell resting_CIBERSORT-ABS	0.548
T cell regulatory (Tregs)_CIBERSORT	0.267	Eosinophil_CIBERSORT-ABS	0.301
T cell gamma delta_CIBERSORT	0.42	Neutrophil_CIBERSORT-ABS	0.951
NK cell resting_CIBERSORT	0.668	B cell_QUANTISEQ	0.001
NK cell activated_CIBERSORT	0.228	Macrophage M1_QUANTISEQ	0.555
Monocyte_CIBERSORT	0.001	Macrophage M2_QUANTISEQ	<0.001
Macrophage M0_CIBERSORT	0.084	Monocyte_QUANTISEQ	0.137
Macrophage M1_CIBERSORT	0.807	Neutrophil_QUANTISEQ	0.809
Macrophage M2_CIBERSORT	0.209	NK cell_QUANTISEQ	0.985
Myeloid dendritic cell resting_CIBERSORT	0.913	T cell CD4+ (non-regulatory)_QUANTISEQ	0.005
Myeloid dendritic cell activated_CIBERSORT	0.322	T cell CD8+_QUANTISEQ	0.14
Mast cell activated_CIBERSORT	0.078	T cell regulatory (Tregs)_QUANTISEQ	0.009
Mast cell resting_CIBERSORT	0.787	Myeloid dendritic cell_QUANTISEQ	0.273
Eosinophil_CIBERSORT	0.307	uncharacterized cell_QUANTISEQ	0.007
Neutrophil_CIBERSORT	0.703	T cell_MCPCOUNTER	0.227
B cell naive_CIBERSORT-ABS	0.004	T cell CD8+_MCPCOUNTER	0.068
B cell memory_CIBERSORT-ABS	0.211	cytotoxicity score_MCPCOUNTER	0.066
B cell plasma_CIBERSORT-ABS	0.558	NK cell_MCPCOUNTER	0.071
T cell CD8+_CIBERSORT-ABS	0.027	B cell_MCPCOUNTER	0.021
T cell CD4+ naive_CIBERSORT-ABS	0.331	Monocyte_MCPCOUNTER	<0.001
T cell CD4+ memory resting_CIBERSORT-ABS	<0.001	Macrophage/Monocyte_MCPCOUNTER	<0.001
Myeloid dendritic cell_MCPCOUNTER	<0.001	Macrophage M2_XCELL	0.424
Neutrophil_MCPCOUNTER	0.993	Mast cell_XCELL	0.254
Endothelial cell_MCPCOUNTER	<0.001	B cell memory_XCELL	0.438
Cancer associated fibroblast_MCPCOUNTER	<0.001	Monocyte_XCELL	0.001

Myeloid dendritic cell activated_XCELL	0.006	B cell naive_XCELL	0.313
B cell_XCELL	0.428	Neutrophil_XCELL	0.775
T cell CD4+ memory_XCELL	0.383	NK cell_XCELL	0.566
T cell CD4+ naive_XCELL	0.039	T cell NK_XCELL	0.606
T cell CD4+ (non-regulatory)_XCELL	0.275	Plasmacytoid dendritic cell_XCELL	0.385
T cell CD4+ central memory_XCELL	0.13	B cell plasma_XCELL	0.271
T cell CD4+ effector memory_XCELL	0.946	T cell gamma delta_XCELL	0.076
T cell CD8+ naive_XCELL	0.798	T cell CD4+ Th1_XCELL	<0.001
T cell CD8+_XCELL	0.273	T cell CD4+ Th2_XCELL	0.403
T cell CD8+ central memory_XCELL	0.149	T cell regulatory (Tregs)_XCELL	0.283
T cell CD8+ effector memory_XCELL	0.494	immune score_XCELL	0.041
Class-switched memory B cell_XCELL	0.609	stroma score_XCELL	<0.001
Common lymphoid progenitor_XCELL	0.135	microenvironment score_XCELL	<0.001
Common myeloid progenitor_XCELL	0.485	B cell_EPIC	0.075
Myeloid dendritic cell_XCELL	0.004	Cancer associated fibroblast_EPIC	<0.001
Endothelial cell_XCELL	<0.001	T cell CD4+_EPIC	0.001
Eosinophil_XCELL	0.018	T cell CD8+_EPIC	0.253
Cancer associated fibroblast_XCELL	<0.001	Endothelial cell_EPIC	<0.001
Granulocyte-monocyte progenitor_XCELL	<0.001	Macrophage_EPIC	0.006
Hematopoietic stem cell_XCELL	<0.001	NK cell_EPIC	0.437
Macrophage_XCELL	0.174	uncharacterized cell_EPIC	<0.001
Macrophage M1_XCELL	0.387		

Table S3. Relationship between the risk groups and clinical characteristics.

Clinical Signature	*p* Value
Gender	0.565
T	0.117
N	0.110
M	0.027
Stage	0.017
Grade	0.721

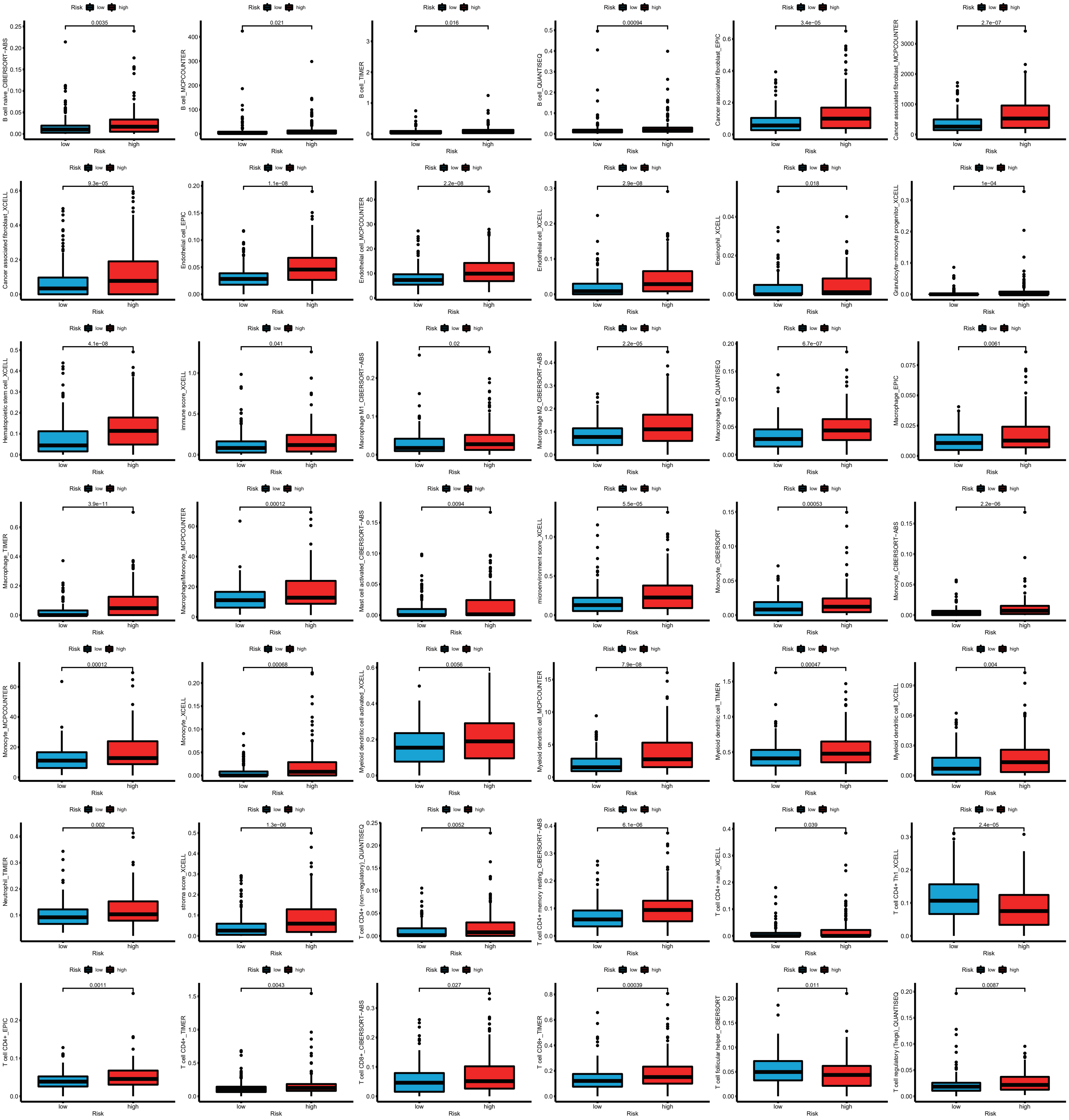

Figure S1. The result of the comparison of immune-infiltrating cells in the two risk group.
